# Analysis of User Behaviour While Interpreting Spatial Patterns in Point Data Sets

**DOI:** 10.1007/s42489-022-00111-9

**Published:** 2022-06-17

**Authors:** Martin Knura, Jochen Schiewe

**Affiliations:** Lab for Geoinformatics and Geovisualization (g2lab), HafenCity University Hamburg, Hamburg, Germany

**Keywords:** Point generalization, Constraints, User behaviour, VGI, Think-aloud study, Punktgeneralisierung, Constraints, Nutzerverhalten, VGI, Think-Aloud-Studie

## Abstract

Volunteered geographic information is often generated as voluminous point data, leading to geometric and thematic clutter when presented on maps. To solve these clutter problems, cartography provides various point generalization operations such as aggregation, simplification or selection. While these operations reduce the total number of points and therefore improve the readability, information preservation could be harmed when specific spatial patterns disappear through the generalization process, possibly leading to false interpretations. However, sets of map generalization constraints that maintain spatial pattern characteristics of point data are still missing. To define constraints that support synoptic interpretation tasks, user behaviour while solving these tasks has to be analysed first. We conduct a study where participants have to perform such interpretation tasks, using a new method that combines think-aloud interviews and techniques from visual analytics. We reveal that the point density of a dataset has the biggest impact on the user behaviour and the respective task-solving strategy, independently from the actual task type executed. Furthermore, our results show that the graphical map complexity only has a minor impact on the user behaviour, and there is no evidence that point data cardinality influences task execution and the solution-finding strategies.

## Abstract (de)

Volunteered Geographic Information wird häufig in großen Mengen und in Form von Punktdaten generiert. Werden die Daten auf Karten dargestellt, können geometrische und thematische Überlagerungen entstehen. Als Lösung bietet die Kartographie eine Vielzahl von Generalisierungsoperationen für Punktdaten an, z.B. Aggregation, Simplifizierung oder Selektion. Während dadurch die Gesamtmenge an Punkten reduziert und somit die Lesbarkeit der Karte verbessert wird, könnten räumliche Besonderheiten des Datensatzes, beispielsweise lokale Extremwerte und Cluster, durch die Generalisierung verloren gehen. Eine Sammlung von Generalisierungsbedingungen (engl. *Constraints*), die die räumlichen Muster der Daten erhält und somit komplexere Interpretationen auch auf Basis der generalisierten Daten ermöglicht, existiert bisher nicht. Um solche Constraints definieren zu können, muss jedoch zunächst analysiert werden, wie sich Kartennutzer verhalten, wenn sie komplexere Interpretationsaufgaben lösen. Wir haben daher eine Studie auf Basis von Think-Aloud-Interviews durchgeführt und das Verhalten der Nutzer anschließend mit Hilfe von visuellen Analysen nachvollzogen. Dabei konnten wir zeigen, dass die Punktdichte der Daten den größten Einfluss auf die Lösungsstrategien der Nutzer ausübt, und dies unabhängig vom konkreten Aufgabentyp. Darüber hinaus konnten wir feststellen, dass die graphische Komplexität der Karte, insbesondere die verwendete Hintergrundkarte, nur eine geringe Auswirkung auf die Ausführung der Aufgaben hat. Ein verändertes Nutzerverhalten bei unterschiedlich komplexen Punktdatensätzen konnte hingegen nicht beobachtet werden.

## Introduction

Volunteered geographic information (VGI), as a special case of the more general web phenomenon of user-generated content (Goodchild 1997), has shown great potential for a variety of scientific and commercial applications. VGI is very often generated as point data, representing a great variety of entities, e.g. points of interest, locations of animal or UFO sightings, spatial information of social media posts, and many others.

VGI data can show an enormous volume as well as semantic and temporal heterogeneity. These aspects can reduce the usability in visual presentation and exploration, introducing geometric and thematic point clutter as well as a decline in rendering performance. The conventional cartographic solution to overcome this cluttering is the process of point generalization, where several generalization operations such as aggregation, simplification, selection or displacement are applied. This cartographic generalization is often done using a constraint-based approach: constraints (i.e., requirements that shall be fulfilled) and related quantitative measures are defined for guiding the generalization process, considering the contradicting aspects of information preservation (preservation constraints) and readability (legibility constraints).

With regard to information preservation, many applications require to maintain specific spatial patterns such as extreme values, clusters or hot spots. If generalization is applied rather general and without considering the actual map use, these patterns could disappear and mislead the user to false results of respective synoptic interpretation tasks that—in contrast to elementary tasks—include pattern identification, pattern comparison or relation seeking (Andrienko and Andrienko 2006). However, until now a set of map generalization constraints to maintain spatial pattern characteristics of point data is missing.

To come up with such constraints, first of all, a better understanding of task-specific user behaviour while interpreting spatial patterns in point data sets is needed. It is of interest to know what information and what priorities are used when users recognize specific spatial patterns during a visual interpretation. Ultimately, it is about preserving this information or these workflows even after generalization. For example, it is of interest in which neighborhood users draw comparisons between point densities to identify clusters and what minimum number of points constitute such a cluster. Accordingly, relative point densities should be preserved even after generalization in a certain environment.

A factor that may have a big impact on user behavior is the *map complexity*. Touya et al. (2016) provide an overview about the numerous concepts and definitions of map complexity in cartography, with most of them agree on the distinction between intellectual and graphical complexity. Intellectual complexity relates to the cognitive process of understanding a map, while graphical complexity relates to the visual perception of the individual map objects (Fairbanks 2006). To address this distinction, it is of further interest to examine if changing the cardinality (i.e. the number of points) of a point data set—and therefore the intellectual complexity of the map—is having an impact on the user behavior while solving synoptic interpretation tasks, as well as changing the background map—and so the graphical complexity.

Furthermore, we are striving for agent-based generalization methods in the future—the findings of the following study will help us to define actions and decisions of agents. In this overall context, our study follows these research questions (RQ):

- *RQ1: Can we identify task-dependent user behaviour while solving synoptic interpretation tasks?*
- *RQ2: How does the cardinality of a point data set influence the user behaviour?*
- *RQ3: How does the graphical map complexity of the background map influence the user behaviour?*

To answer these questions, we introduce relevant literature (Sect. 2) and conduct a study where participants have to perform specific interpretation tasks using point data visualizations (Sect. 3). Based on the results of our study (Sect. 4), we identify the task-dependent user behaviour (Sect. 5) and conclude our article (Sect. 6).

## Previous Work

In their work on display clutter and respective measurement techniques, Moacdieh and Sarter (2015) refer to display clutter as a very dense or even overlapping display of entities on a screen. Depending on the application domain, these entities can vary widely: words and graphics on webpages, icons on an airplane cockpit display, or—with respect to cartography and VGI points—symbols or markers on a map.

To overcome these point clutter problems on maps, a variety of fundamental generalization operations such as aggregation, simplification, selection, displacement or spatial distortion can be applied (e.g., McMaster and Shea 1992; Hake et al. 2002; Slocum et al. 2009). In *aggregation* operations, point clusters based on geometric and/or semantic criteria are identified and replaced by aggregator markers. The cluster initialization method (like random, k-means, center-, grid-, or Voronoi-based) thereby influences the final result (Burigat and Chittaro 2008; Yan and Weibel 2008). *Simplification* operations reduce the source points based on geometric criteria such as minimum distances between points (Slocum et al. 2009). *Selection* operations also perform point reduction, but now based on semantic criteria, e.g. using respective information filtering methods (Huang and Gartner 2012) or scale-dependent parameters (Gröbe and Burghardt 2021) to determine which points are preserved. For a better distribution of points, *displacement* operations can be applied (Mackaness and Purves 2001). *Spatial distortion* changes the geometric arrangement of points by maintaining topology (Ellis and Dix 2007), e.g. through pixel-based (Keim et al. 2004) or point density equalizing distortions (Bak et al. 2009).

Selecting and/or combining adequate generalization operations can be done using a constraint-based approach, with constraints representing the conditions a generalised map should satisfy (Harrie and Weibel 2007). Beard (1999) classified these constraints into aspects related to position, topology, shape, structure, function, and legibility. For practical and evaluation purposes, constraints must have related measures, which were grouped by Mackaness and Ruas (2007) into internal and external, as well as into micro, meso and macro categories. If defined in a complete, precise and measurable manner, a constraint-based approach is able to guide the overall generalization process and applied to automated generalization using different techniques (Stoter et al. 2014; Duchêne et al. 2018).

For the interpretation of spatial patterns in a generalized map, there are two potentially contradictory aspects to consider when using constraint-based approaches: information preservation and legibility. *Information preservation* constraints are normally described in terms of geometrical object-specific measures, e.g. the preservation of building area and angularity before and after generalization (Harrie and Weibel 2007). Until now, less attention is paid to the preservation of existing patterns in the dataset, such as clusters or extreme values. Legibility constraints on the other hand ensure that these patterns are still readable by the users, avoiding any spatial conflicts and using only objects which are large enough, but not too detailed. Stigmar and Harrie (2011) developed analytical *legibility* measures for topographical maps, such as a number of vertices, object line length, local density, proximity indicator, and degree of overlap. But despite the developments for single constraint definitions, complex measures which support synoptic interpretation tasks are still difficult to define (Stoter et al. 2014; Burghardt et al. 2007).

## Qualitative Study

We conducted qualitative studies to learn more about user behaviour dealing with VGI point data. First, we performed a preliminary study to identify relevant tasks typically performed on point data and to test the suitability of the chosen point data sets. In the main part of the study, we conducted a new type of think-aloud interviews and examined user behaviour while solving different synoptic interpretation tasks.

### Task Definition and VGI Datasets

In our studies, we used the task typology defined by Andrienko and Andrienko (2006). The authors divide tasks into elementary ones, which refer to individual elements of the data, and synoptic tasks, which take a subset of the whole dataset into account. Tasks in both categories can be distinguished into direct and inverse lookups, direct and inverse comparisons, and relation-seeking.

To examine the different types of tasks, we created various maps using VGI point data sets from different sources:

- Italian restaurants in the inner city of Hamburg, Germany, retrieved from Open Street Map via the Overpass API.
- Pictures tagged either with “Cristo Redentor” or “sugarloaf” and provided with a photo location in Rio de Janeiro, Brazil, retrieved via the Flickr API.
- Pictures provided with a photo location in the Lüneburger Heide, Germany, retrieved via the Flickr API
- Bird sightings in the Nature Park Lüneburger Heide, Germany, retrieved from (1) the eBird Basic Dataset and from (2) the iNaturalist database.
- Antelope sightings at Kruger National Park, South Africa, retrieved from the iNaturalist database.

For all datasets retrieved from the Flickr API, we created smaller subsets for examining the influence of the point cardinality on user behaviour. To preserve the initial clusters, we reduced the number of points incrementally and analysed after each step if all clusters are still identified by the HDBScan algorithm.

### Preliminary Study

Goals of the preliminary study were to identify and select relevant interpretation tasks for our datasets, to get a first impression of the user behaviour and confidence while solving these tasks, and to evaluate the suitability of the datasets for the purpose of overall study objectives. Beyond that, we wanted to find manually defined patterns in our datasets, which we used during the encoding process of the following interviews.

The preliminary study was conducted as a postal questionnaire with 25 participants, all of them professionally working with maps. For the first part of the study (Part A), a printed map of the Italian restaurants in the inner city of Hamburg, Germany, was provided. The dataset was also used for the second part (Part B) but is now extended with randomly generated information about the price level of each restaurant, which was symbolized with different colours. The map for the third part (Part C) showed the distribution of sightings of four different antelope species in the Kruger National Park, indicated with different colours. Based on the aforementioned task typology, we generated three elementary and nine synoptic tasks, and increased task difficulty (elementary vs. synoptic tasks) and complexity (mono- vs. multi-categorical data) from part to part (see Table 1). After the last question of each part, the participants had to rate their certainty while answering the questions about the map.

**Table 1:** Tasks of the preliminary study

ID	Task	Purpose
**Part A**	**Italian restaurants in Hamburg**	
1	Count restaurants in a given district	Examine if (elementary) direct lookup tasks are suitable for mono-categorical point data sets
2	Mark all clusters of restaurants on the map	Examine if (synoptic) pattern search tasks are suitable for mono-categorical point data sets
3	Compare the number of restaurants in two districts	Examine if (elementary) direct comparison tasks are suitable for mono-categorical point data sets
4	Describe spatial patterns of restaurants in a given district	Examine if (synoptic) pattern definition tasks are suitable for mono-categorical point data sets
5	Rate certainty while answering the questions	Obtain user confidence while working with the map
**Part B**	**Price level of Italian restaurants in Hamburg**	
6	Identify district(s) with many high-priced restaurants	Examine if (elementary) inverse lookup tasks are suitable for multi-categorical point data sets
7	Identify district(s) with all the different price levels	Examine if (synoptic) direct comparison tasks are suitable for multi-categorical point data sets (focus: same attributes over different references)
8	Compare price levels of districts to that of a given one	Examine if (synoptic) relation-seeking tasks are suitable for multi-categorical point data sets (focus: specified attribute behavior of a reference)
9	Rate certainty while answering the questions	Obtain user confidence while working with the map
**Part C**	**Antelope sightings in the Kruger National Park**	
10	Mark clusters of sightings on the map	Examine if (synoptic) pattern search tasks are suitable for multi-categorical point data sets
11	Identify the most frequent antelope specie	Examine if (synoptic) direct comparison tasks are suitable for multi-categorical point data sets (focus: different attributes over same references)
12	Identify the least frequent antelope specie	
13	Compare positions of given antelope species	Examine if (synoptic) inverse comparison tasks are suitable for multi-categorical point data sets
14	Find similar patterns in same regions between different antelope species	Examine if (synoptic) relation-seeking tasks are suitable for multi-categorical point data sets (focus: same attribute behavior over different references)
15	Rate certainty while answering the questions	Obtain user confidence while working with the map

**Fig. 1:**
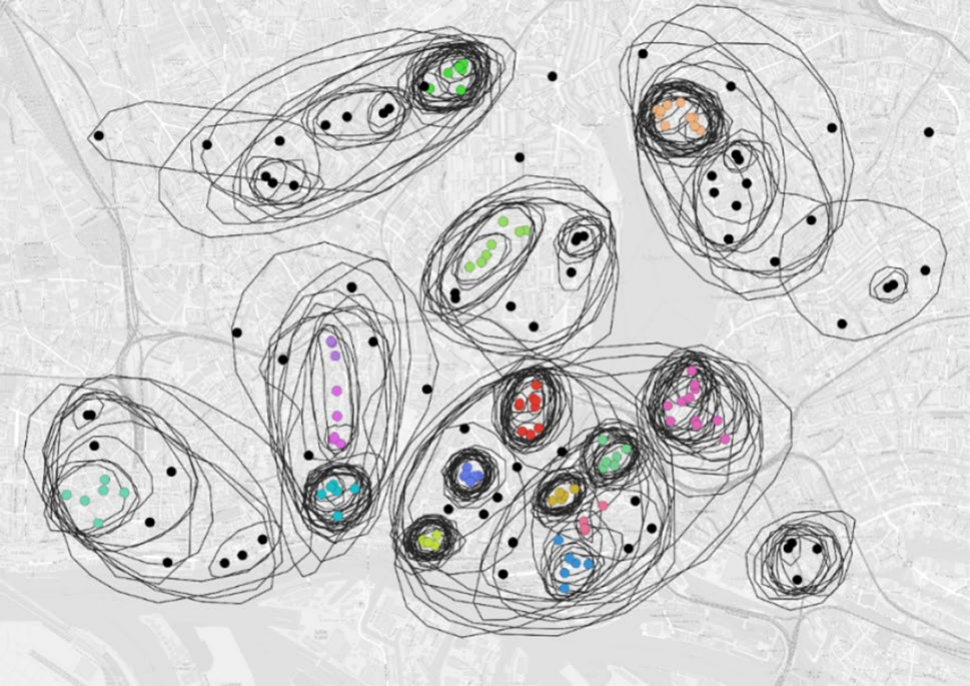
Comparison between clustering by study participants (black lines) and HDBScan clustering algorithm (coloured dots; black dots were not assigned to a cluster)

Elementary tasks of lookups and comparisons were very well understood and solved by the participants. In synoptic pattern identification tasks, participants were able to characterize patterns with their own words, often using additional information presented on the map. Furthermore, they argued with local pre-knowledge such as street names or landmarks and marked reasonable clusters on the map. As shown in Fig. 1, most of these clusters were also identified by the HDBScan Clustering algorithm. Synoptic comparison tasks had the best success rate within the whole questionnaire, with correct answers ranging between 88 and 95%. The answers for relation-seeking tasks showed greater variability, with the participant’s solving confidence being lower than before. Because most of the participants answered all questions, we argue that the answer variety was caused by different solution strategies.

To limit the scope of our study, we decided to focus solely on synoptic tasks in the following main study. We wanted to further investigate the user behavior—and, therefore, identify potentially different solution strategies—while having tasks for each of the synoptic subtypes (pattern identification, pattern comparison, relation-seeking).

### Main Study

**Fig. 2:**
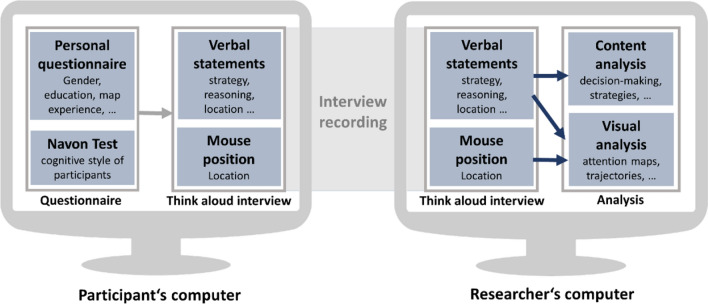
Technical scheme of the interview and the analysis process

The main study focused on the overall objective—the analysis of user behaviour while solving synoptic interpretation tasks. We also wanted to find out whether the cardinality of the point data set, as well as the graphical map complexity of the background map, have an influence on the task-solving strategy of the participants. Figure 2 gives an overview of the design of the main study.

#### Study Design

##### Method

Originally, we planned an eye-tracking study, but due to the ongoing COVID-19 pandemic, the study design changed to an online study. First, we sent a questionnaire to the participants and asked for their gender, age, education, their experiences and skills with maps and their geographic knowledge (see left column in Fig. 2). Furthermore, we invited the participants to perform a Navon test to define their cognitive style via the open-source software library PsyToolkit.^1^ After a short introduction, they had to answer 50 questions within a short time using a provided web interface and fill in their results in a table provided at the end of our document.

The second part of the study was held via a Zoom meeting and can be described as a Think Aloud interview, a qualitative research method where the user is requested to vocalize her or his thoughts while answering a question or solving a task (Eccles and Arsal 2017). After a short introduction to the method, participants had to share their screen during the interview. We recorded their screens and voices, with only the maps and their mouse symbol visible to us (see the centre of Fig. 2). After the test, we anonymized all related data files, so backtracking from the interview to the participant was not possible anymore.

We chose a between-subject design for our study, where each participant had to solve five tasks, and examined the user behaviour through a set of ordered task-solving actions. Every task was introduced with an explanation about the map, the underlying data and the task, and participants could decide when to start. While solving the task, each participant described her or his behaviour and answered questions from the interviewer. With that, the method is a mixture of concurrent and retrospective Think-Aloud as described by Häder (2006). Although we guided the interviews by predefined questions, we waited with our first question until the participants—in their opinion—seemed to finish the respective task (*concurrent* Think Aloud). The interview guidelines included a list of questions regarding the different actions a participant made to solve the task, their order, the decision-making process, and if special characteristics of the map had influenced their strategies. With the guidelines, we made the interviews comparable and ensured that each question was answered by the participants, either directly while thinking aloud, or on request afterwards (*retrospective* Think Aloud).

##### Participants

Twenty-one people (7 female and 14 male) participated in the main study voluntary, with none of them already participating in the preliminary study. All of the participants were either students or postgraduates from the HafenCity University Hamburg, Germany. Regarding geographical knowledge, two participants rate themselves as experts, 15 participants as advanced, and four as a layman. All participants rate their experience in using maps as either average or expert. The questionnaire further revealed that two of the participants use satellite data every day, eleven often and eight rarely.

Nineteen of the twenty-one participants provided their results of the Navon test. The Navon test is a method to define the cognitive style of a person, which can be holistic or analystic. Holistics tend to focus on the large-scale patterns (“Forests before trees”; Navon 1977), while analytics tend to examine individual parts and their connections. Because the perception of global features is a crucial part of solving synoptic tasks, our hypothesis was that user behaviours significantly differ between analytic and holistic cognitive types. Using the results provided by PsyToolkit, we followed the same practice as Opach et al. (2019). In the end, we identified seven participants each with an analytic or holistic cognitive style and five participants which were neutral following this definition.

##### Study Material

We used five different maps on four locations for our study (see Table 2), and randomly assigned the map variations to the participants. Point colour was used to differentiate between data sources (task 1), tags (task 2), antelope species (tasks 3 and 4) and the price levels of restaurants (task 5). We use the same scale within variants of the same location, but different scales for each location to ensure that the resulting user behaviour identified in this study is not bound to a certain scale. All but the last map was provided without any information or labels hinting at the location of the map. On request, nobody identified the locations correctly, so we can state that all locations were unknown to the participants. We varied the cardinality of the point data sets and the background map and calculated the respective map load with the graphic map load measuring tool (GMLMT). We use the map load measure of GMLMT as the indicator for graphical complexity in our study, which uses edge detection to measure the graphic map load as the amount of visible structures in a map (Barvir and Vit 2021). As shown in Table 2, we decided to use background maps with rather low map loads, as we want to make sure that the confounding impact of the most complex background map is still small enough to allow a straightforward interpretation of the respective point data sets.

##### Tasks

As a result of the preliminary study, we decided to focus on synoptic tasks, with increasing complexity (task numbers correspond to the map identifiers used in Table 2):

1. *Pattern identification*: Describe the distribution of points representing bird sightings and photos taken at the Nature Reserve.
2. *Direct comparison*: Compare the distribution of points representing photos with tags of two landmarks.
3. *Inverse comparison task*: Describe the relative positions of sightings of two given antelope species.
4. *Relation-seeking between different attributes within the same areas*: Identify other antelope species which have similar patterns of sightings compared to the two species of task 3.
5. *Relation-seeking* *between the same attribute within different areas*: Identify districts which have a similar price level to that of a given district.

#### Analysis

First, we cut the interview recordings into smaller clips of single tasks and sort out all other parts (e.g. introduction to the method, discussions between tasks). Then we encoded each clip twice (see right column of Fig. 2): For the content analysis, we wrote the participants’ reasoning down while solving the task and answering the predefined interview questions. For the visual analysis, we implemented the encoding system described in Knura and Schiewe (2021). It allowed us to encode the content and the focus location on the map for every second of the clip, using the statements of the participants and their mouse cursor as indicators. We defined seven content categories: (1) task description, (2) interviewer’s question, (3) participant’s question, (4) non-task-related discussion, (5) legend, (6) background map and (7) data. Data-related content referred either to the whole dataset or to one of the attributes which we encoded as subclasses for category (7), e.g. we had a subclass for each of the antelope species in task 3. For encoding the location, we used the manually marked clusters from the results of the preliminary study (see Fig. 1) for tasks which use the same data (tasks 3 and 4). Otherwise, we define clusters either by applying the HDBScan algorithm (tasks 1 and 2) or based on the dataset itself (city districts for task 5).

From the results of the encoding process, we created visualizations using techniques from eye-tracking analysis (Andrienko et al. 2012), e.g. flow maps, attention maps or map displays of trajectories. We are aware that we cannot reveal the actual eye movements consisting of fixations and saccades with our technique, but we can reconstruct the task-solving approach of each participant. To distinguish it from actual eye-tracking visualizations, we will use the terms *focus map* and *focus trajectory* when referring to the results of our encoding process. For further analysis and clustering different strategies between the participants, we used the MultiMatch method proposed by Jarodzka et al. (2010), a vector-based approach originally developed to compute scan path similarity for eye-tracking data.

In general, we did not use viewing time as a dependent variable in our study, as this factor heavily depends on the users’ capabilities to express their thoughts comprehensively, which is of course an essential part of think-aloud interviews.

## Results

This section presents the results derived from both the content analysis and the visual analysis—sorted according to the different tasks. For the visual analysis, all figures are created with the Python library matplotlib, based on the encoding data obtained as described above.^2^

### Pattern Identification Task

**Fig. 3:**
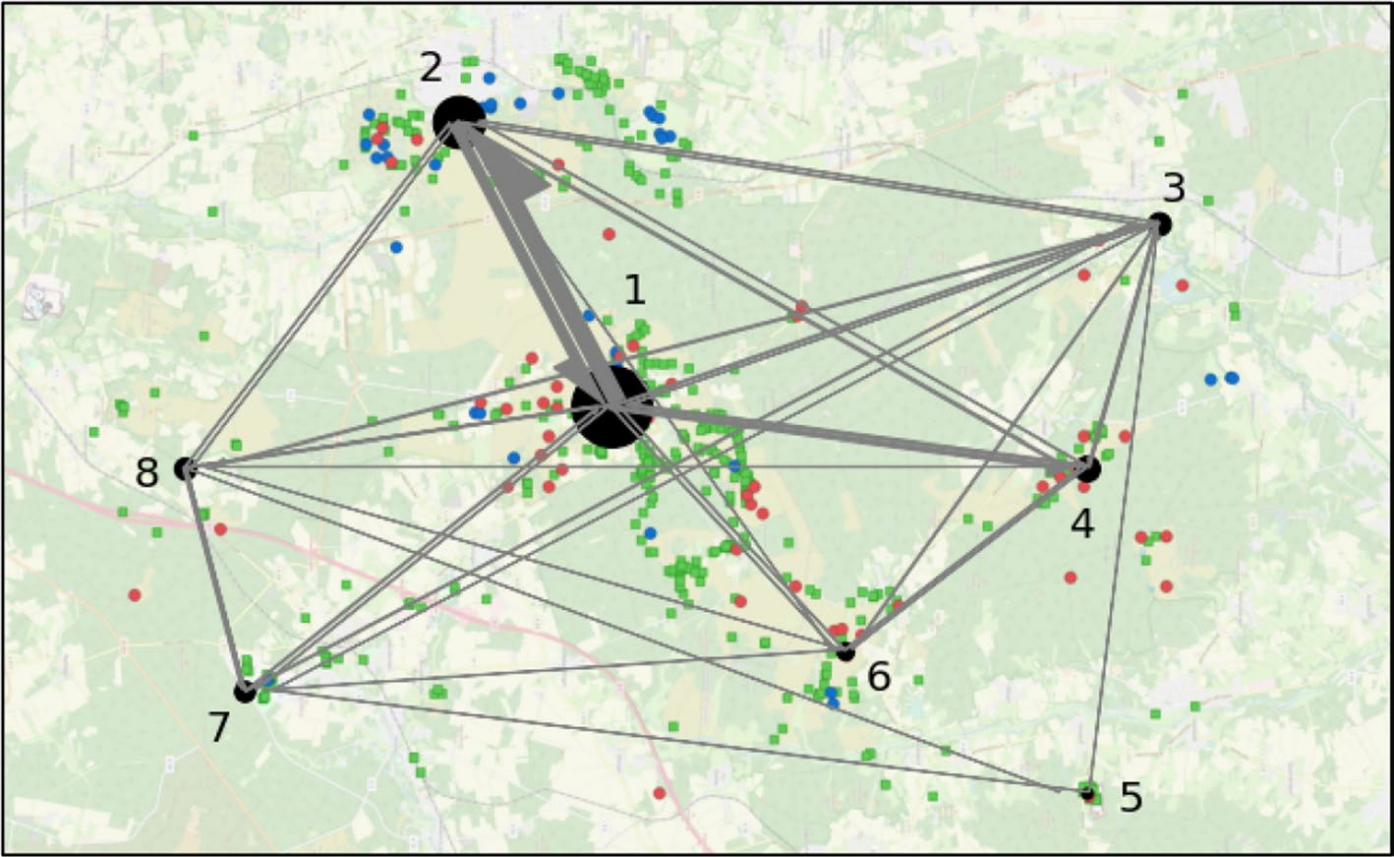
Focus map of all participants using predefined clusters for task 1. The size of the black points relate to the cumulative frequency participants focus on the cluster, while the arrow sizes show the frequency of the participants moving between the respective clusters

For the first task of the study, participants had to identify patterns on a map with point data derived from Flickr, eBird and iNaturalist (see Sect. 3.1), while the cardinality of Flickr points and the background map was varied between them (see Table 2). Although dot colours of red, green and blue were used, there were no specifications made on which colour to start with. Independently from the category they focus on, 18 of the 21 participants identified the biggest cluster in the centre of the map (cluster 1) first (Fig. 3). The other three started with the cluster in the north (cluster 2), and two of them moved to Cluster 1 thereafter, so only one participant identified cluster 1 as the third choice. While the frequently visited path from 1 towards 2 can be explained by the high amount of participants starting in this order, the reverse path is mainly caused by comparisons between the clusters.

**Table 2:** Map details and variants for each task

Task	Var.	Location	Point data sets (point cardinality)	Basemap	Map load (%)	Participants
1	a	Lüneburger Heide	**Flickr (600), ebird (50), iNaturalist (50)**	**Google Satellite**	15.1	7/21
	b		*Flickr (200), ebird (50), iNaturalist (50)*	**Google Satellite**	14.9	9/21
	c		**Flickr (600), ebird (50), iNaturalist (50)**	*OSM*	7.7	5/21
2	a	Rio de Janeiro	**Flickr (1000)**	ESRI Grey	6.7	9/21
	b		*Flickr (500)*		6.3	12/21
3	a	Kruger National Park	iNaturalist (530)	*Bing Maps*	5.2	7/21
	b			**Google Maps**	12.8	7/21
	c			*Stamen*	5.7	7/21
4	–	Kruger National Park	iNaturalist (220)	Bing Maps	5.2	21/21
5	–	Hamburg	OSM (170)	OSM	28.6	21/21

Variations for tasks 1–3 were randomly split between the participants with a distribution as specified in the last column

Map variations with high point cardinality and graphical complexity are marked in bold, variations with low cardinality and complexity are marked in italic

In general, most of the participants verbally differentiated between the “dense” areas of clusters 1 and 2, and “sparse” areas. When talking about “dense” or “density”, participants referred—throughout the whole study—to areas with numerous points of close proximity, with the proximity seeming more important than the cardinality of the cluster. Still, both aspects were important, because as a result of our variations of the point cardinality, participants tended more towards cluster 1 when there was a higher amount of points on the map. Of the randomly chosen 12 participants facing the variant of 600 points originated in the Flickr dataset, all start with the biggest cluster 1.

Beside the identification of clusters, the point distribution can also be related to the given background map, because all clusters are located on open fields and close to walking trails. Only five participants mentioned the background map on their own, while the other participants described the relation between background map and point distribution not until a respective request from the interviewer came up. Apart from the statements of the participants, the focus maps of users differed between the different backgrounds, as there was much more interaction with the majority of the sparse clusters from the participants working with the less complex background map (here: OSM).

### Comparison Task

The map for task 2 visualized the point locations of Flickr photos taken in Rio de Janeiro which were either tagged with “Cristo Redentor” (orange dots) or “Sugarloaf Mountain” (blue dots), and participants had to compare the two-point distributions. We suppose that the landmarks were present on the majority of these respective tagged photos, so after the initial task, participants guessed the exact location of each landmark based on the potential line of sights.

**Fig. 4:**
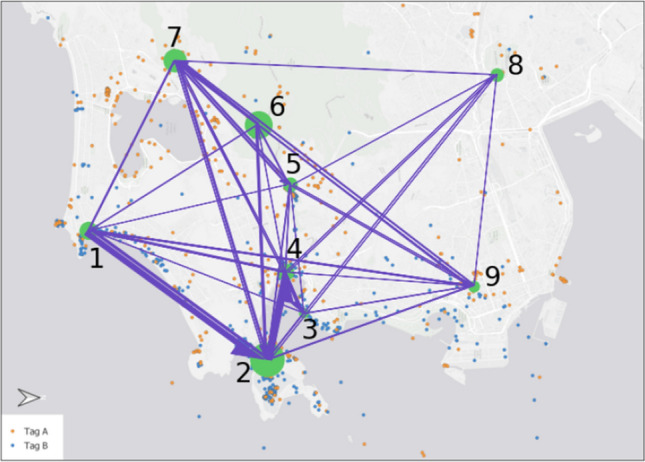
Focus map of task 2 based on predefined clusters; note that North is on the right side of the map

Even though the orange point set was the first listing in the legend, 18 out of the 21 participants started with the description of the blue points, following the coastline from cluster 1 (see Fig. 4) to the bigger peninsula in the east (cluster 2) und further west into the small bay (cluster 4). After that, strategies differed between the participants, with some heading back east and others following the coastline further north (cluster 9) before focusing on the orange point set in the west (clusters 6 and 7). We argue that this prioritization of the blue points is mainly the result of the used colours and their respective saliency, and, furthermore, raised by the particular cluster shape following the coastline. Nevertheless, the hierarchy of clusters within each category was the same, starting with the densest cluster to the least dense.

**Fig. 5:**
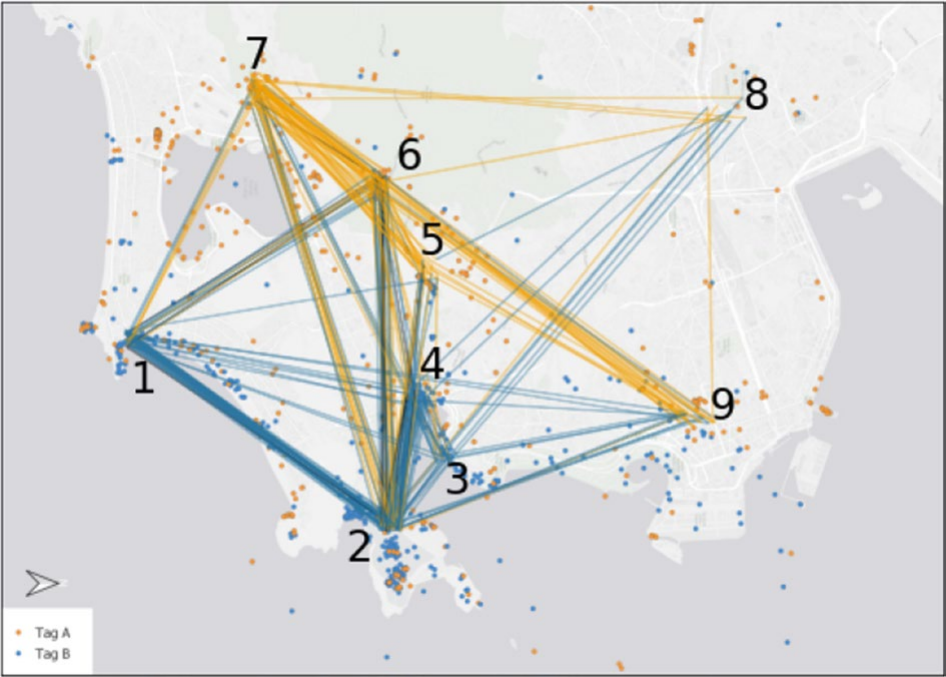
Focus trajectory paths of the participants, the colour of the arrow indicates the colour of the tag they are focusing on while transitioning

In contrast to the first task, participants referred frequently to the background map during their reasoning, describing the coastline, the harbour, built-up areas, and the mountainous region in the west. Again, the differentiation between dense and sparse areas was highlighted by most of the participants, this time in combination with the different colours. As shown in Fig. 5, clusters of blue points were more identified along the coastline and stated as dense, while the orange point clusters were more often stated as sparse and in the hinterland of the city.

### Inverse Comparison Task

For the third task, participants had to describe the relative positions of the sightings of two different antelope species on a map of Kruger National Park, with one specie (orange) sighted more frequently in the northern part, and the other (blue) more southwards.

**Fig. 6:**
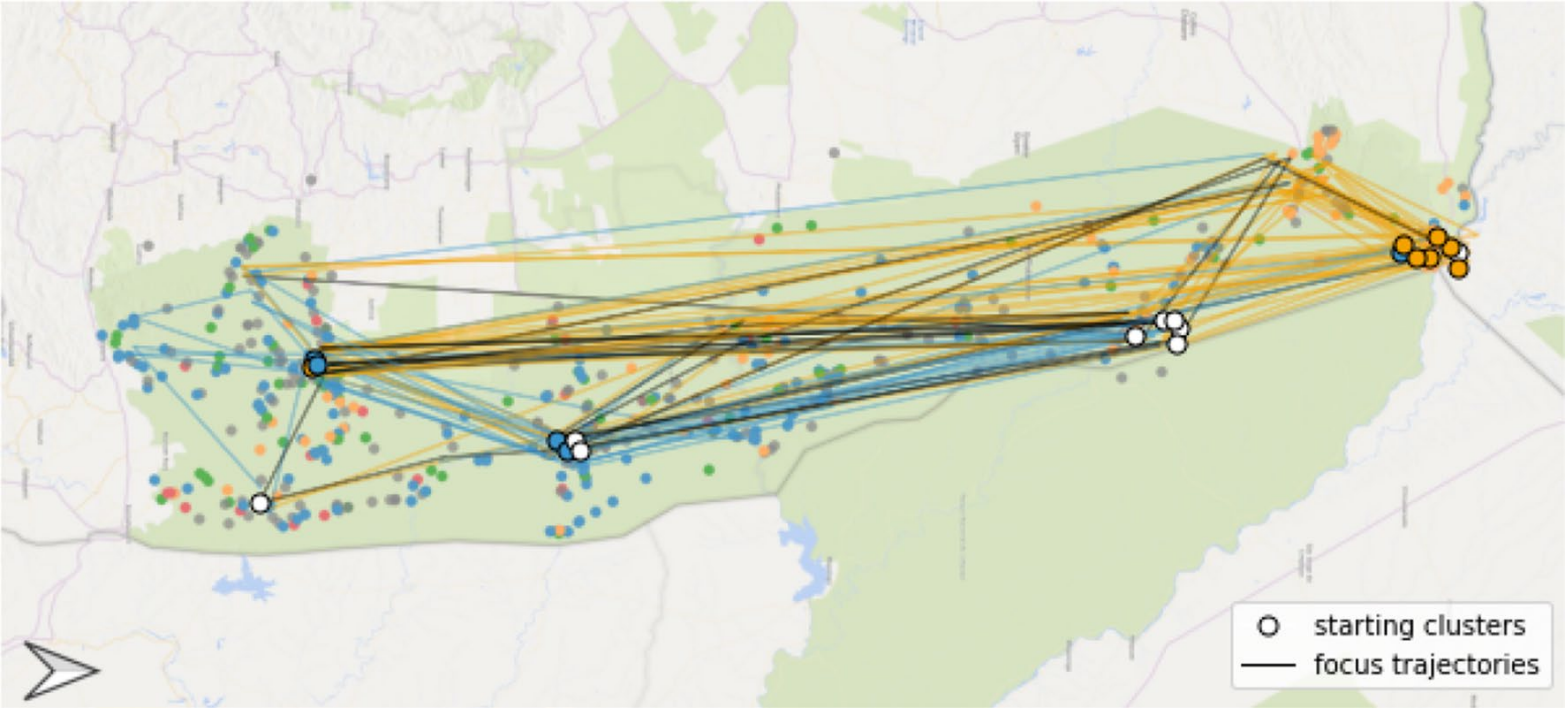
Focus map of task 3. Colours of circles and lines correspond to the antelope specie they relate to. White colour means participants start without focusing a particular antelope specie

When comparing the distributions, participants focused mainly on two aspects. First, they compared the location of the clusters of each antelope specie. Most of them described the north–south differentiation explicitly or implicitly correct, which can also be seen in the colour distribution in Fig. 6 where orange is much more described in the north and blue in the south. Twelve of the 21 participants also identified regions where both antelope species appear simultaneously. Second, they analysed the density of the different coloured point distributions. As before, they differentiated between areas with high point density described as “dense”, and areas with low density, respectively, using words and phrases such as “sparse”, “not crowded”, “spread out” or “scattered”.

In general, the task-solving behaviour of the participants was very heterogeneous for this task. In contrast to the previous task, this time more participants started with the orange category, which again can be explained by the higher saliency from the background map. But other than in task 2, there was no particular starting cluster the majority of participants chose (see Fig. 6). They started either on one end of the north–south dichotomy or with a line-shaped cluster in the centre, which was also described by many participants as unique and eye-catching. From there on, 17 of the 21 participants switched at least three times between the northern and southern half of the map, but with no dominant north–south trajectories between two specific clusters.

Furthermore, we varied the background map for this task. While this had no effect on the overall task-solving strategies, the subgroup using the stamen background map focused much less on a central cluster close to a river. We argue that the characteristics of the other two background maps guided the users to this cluster, while the low visibility of the river hamper this effect on the stamen map.

### Relation Seeking Task Between Different Classes

For task 4, participants had to find similar distributions of other antelope specie sightings compared to the ones in task 3. The overall strategy of the participants was quite similar between all. 14 of them focused on the distribution of the black points from the start, arguing that these black points stand out against the rest due to their colour saliency and the low density in the areas the points appear. After choosing their first antelope specie, 18 of the 21 participants described the point distributions of at least two other species, before making their decision. In comparison to the previous task, there were two inherent differences, which can be explained by the repetition of the map topic and the relation to the task before: First, the background map was not part of the reasoning anymore. Second, participants generalized much more in their statements by just distinguishing between distributions appearing “more in the South” or “more in the North”, as about one-third of all statements referred to these broad areas. Apart from that, the high number of transitions between the northern and southern parts of the map was still present during this task, and again with no dominant connection between two certain clusters.

### Relation Seeking Task Between Spatial Areas

For the last task, participants had to find city districts that have a similar price level of Italian restaurants (marked as points) compared to a reference district.

Other than before, the first part of the task-solving strategy was similar between all participants, as they searched for the reference district on the map, and defined their individual comparison criteria by describing the distribution of different price levels in this district. After that, they moved over the map to identify the most suitable candidates of similar districts and made their decision based on the comparison criteria and the best candidates they have found.

The content analysis of the statements revealed that there were four different comparison criteria used by the participants, with 14 of them using at least two of these criteria during the task-solving process. The comparison criteria regarding the reference district were an overall number of points (named 13 times), point proportion between the different price level classes (11 times), mean price level (10 times), and absence of the highest price level class (10 times).

**Fig. 7:**
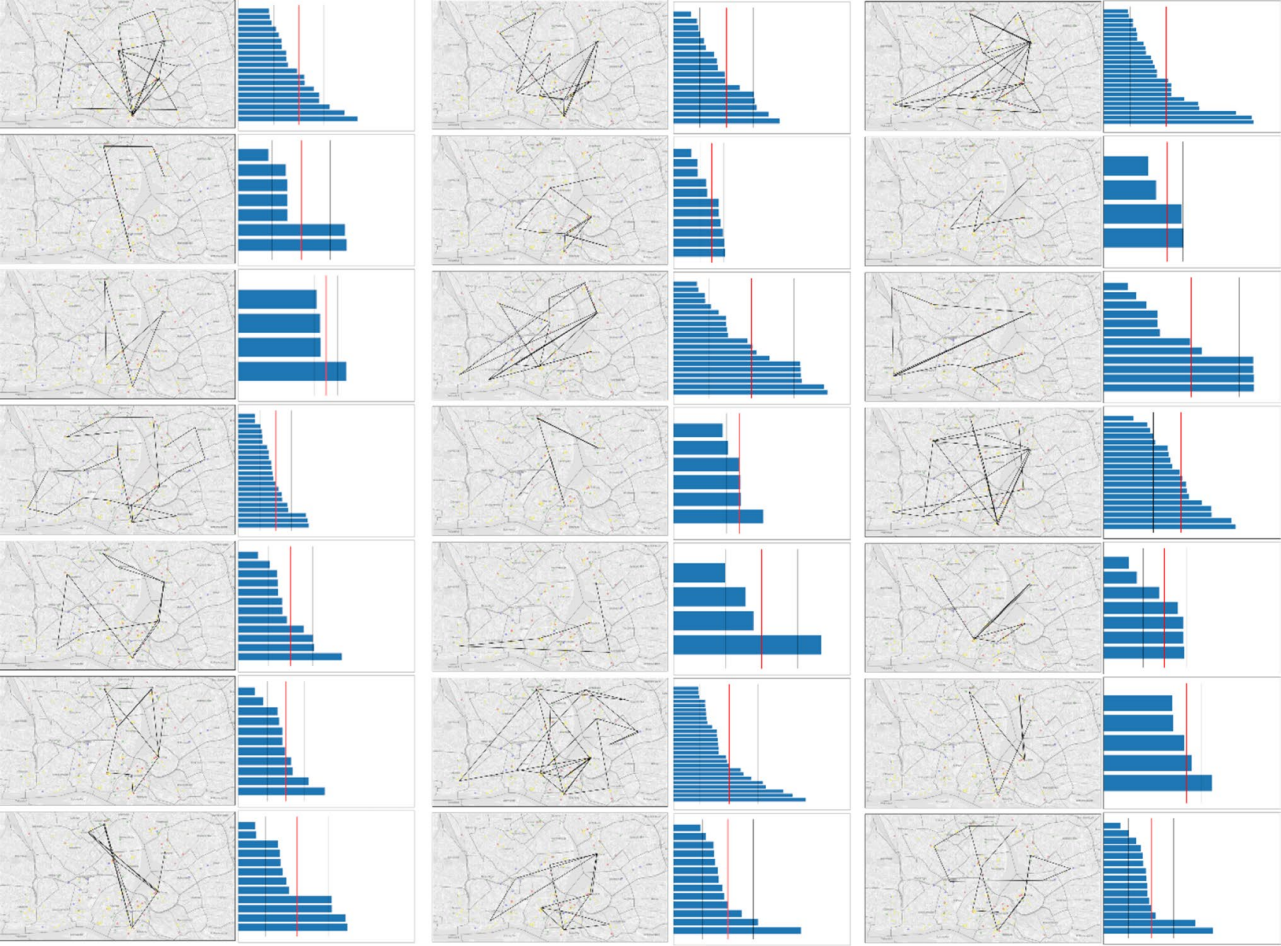
Focus trajectory paths and the corresponding sorted path lengths in map pixels of each participant. Red line indicates the mean path length

After defining the comparison criteria, the next step was to search for suitable candidates. Figure 7 shows all trajectories of the participants while moving over the map. Looking at the trajectories and the corresponding sorted path lengths, two search strategies can be derived: Firstly, some of the participants moved stepwise through the map, passing every district with short distances in between, leading to a large number of short paths (*sequential* strategy). The second approach represents the opposite characteristics, with more of the longer trajectory paths: Starting from the reference district, these participants moved less strategical through the map, focusing only on selected districts, and moved back to the reference district several times (*star* strategy).

**Fig. 8:**
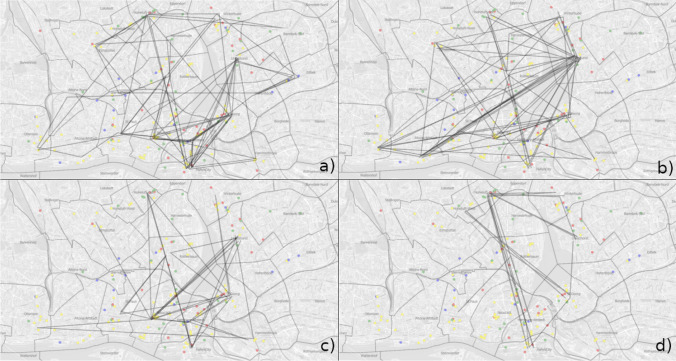
Cumulated flow maps for each search strategy approach: **a** sequential, **b** star, **c** central cluster focus and **d** north cluster focus

Within participants not following one of the approaches described above, we find two more search strategies using the MultiMatch algorithm for position similarity: One group of participants tended to move towards the area of the map with the highest point density (*central cluster* strategy), the other group focused more on a cluster in the north of the map and much less on the most-dense areas further south (*north cluster* strategy). Figure 8 shows the cumulated flow maps of all participants belonging to the respective search strategy group. Nevertheless, we find no evidence for any correlation between the defined comparison criteria of users and their search strategies.

### Influence of Cognitive Type and User Characteristics on Behaviour

According to the answers of the questionnaires and the Navon test, we subdivided our participants into groups and examined if we find significant differences in the user behaviour for each of the five tasks. To compare the groups, we created focus maps of differences for the cognitive types, gender, and academic degree, and looked for notably statements in the content analyses. There was no clear difference between the groups in any of the tasks. On the other side, due to the small number of participants in our study, and the fact that we could only assign 14 of the 21 participants to a cognitive type, we have no evidence that user characteristics did not have an influence on the behaviour. We will address this aspect in a future study.

## Discussion

In the following, the aforementioned results for individual tasks will be summarized and discussed to answer the overall research questions of this study.

### Strategies Solving Synoptic Interpretation Tasks

We analysed the participants’ overall task-solving strategies focusing on the behaviours for selecting a start position and the following process of obtaining information and decision-making. Regarding the starting position, the density of points—as a combination of proximity and cardinality—in a cluster seems to be the most important factor (as observed in tasks 1 to 4), followed by point colour (see tasks 2 to 4). While the density of points is a data-related factor, we introduced the second factor with our colour choice and the respective contrast and visual saliency. Beside these two aspects, a unique cluster shape was the only other characteristic attribute attracting participants from the beginning. For the fifth task, all participants started their task-solving by searching for the explicitly given reference district.

The point density of clusters was also the most important factor during the following process of obtaining information. If there was more than one alternative category to deal with—as in the tasks 1, 3 and 4—the one with a denser cluster was described and analysed earlier and more often, while sparse distributions were ignored by many participants. For comparison tasks, density was also the main evaluation measure that participants used to describe and distinguish clusters, and an important factor influencing their respective search strategy in task 5.

In general, we can distinguish different task-solving strategies; however, these strategies do not differ significantly between the different task types. Within our study focusing on VGI point data, we can state that the point density has the biggest impact on the user behaviour during the task-solving process. This answers our research question **RQ1**.

### Influence of Point Data Cardinality

Concerning the influence of the point cardinality on the task solving process, we varied the amount of points on the map for both tasks 1 and 2, and compared the respective user behaviour for each step of the solving process. As described in Sect. 4.1, the point cardinality had an impact on the first cluster identified in task 1, as all participants who dealt with the higher cardinality started with the biggest cluster. In task 2, there was no significant difference in the starting positions between the two variants. We explain this with the different map topics. Task 1 shows bird sightings and photo spots in an *open* landscape, so adding more bird observations and photos introduces more locations and possible clusters on the map. Task 2 shows much more concentrated photo locations in a city, as the two landmarks cannot be seen from everywhere in the city. Therefore, increasing the size of the dataset primarily adds more photos to the existing sightseeing spots. However, for both tasks, the task execution strategy itself and the solution-finding were not influenced by the point cardinality. In summary, we can state that user behaviour and solving strategies did not change significantly with higher point cardinality, which answers our research question **RQ2**.

### Influence of Background Map

The second variable we changed within our study was the graphical complexity for tasks 1 and 3. As shown for task 1, the vast majority of the participants referred to the background map only on request. However, as the focus maps differ slightly, an implicit effect on the task execution process is possible. Between the three background maps used in task 3, the only significant difference was the low frequency of participants focusing on the central cluster at the river while using the stamen background map. As stated in Sect. 4.3, we explain this with the low visibility of the river signature.

In general, we see both an implicit and explicit influence from the background map on the participants’ reasoning for all the five tasks: On the one hand, participants explained the cluster characteristics referring to the background map. On the other hand, frequently identified clusters were implicitly supported by background map characteristics, even if not explicitly mentioned in the statements during the interviews. Although the background map can be part of each step of the task-solving process, we found no evidence that the graphical complexity of a map influences the overall strategy to solve the given task.

The only task execution strategy we identified throughout our study which is clearly defined by the background map is the sequential strategy during task 5 when the order of districts was defined by neighbourhood and not by point density. Answering our research question **RQ3**, we state that the graphical complexity in general, and the background map in particular, have an implicit and explicit influence on the participant’s reasoning, but do not change the overall task-solving strategy.

### Recommendations for Constraints in Map Generalization

Both preservation and legibility constraints in map generalization have to address point density in the first place. In the following, we provide recommendations for defining constraints that shall be used for guiding the point generalization process.

#### Preserve Pattern Proportions

For the preservation of spatial pattern, two aspects have to be considered. First, the proportion of points between the different patterns, as well as between dense and sparse areas, has to be retained. As the solving strategies for comparison and relation-seeking tasks show, it is of major importance that generalization operations fulfil constraints by preserving the proportion of objects under consideration. For instance, in task 5 of our study, the individual pattern definition depended on the user’s interpretation of *similarity*, and the criteria most frequently used was the total number of points within a district. Moreover, our results reveal that the point density of clusters or classes was the main attractor to the participants, and the point density predominantly defined the order of areas recognized. A potential solution for this demand is constraints preserving the ranking of densities between focus areas.

The second aspect is the proportion of points of different classes within an area. For all tasks, the participants compared the characteristics of different clusters, classes or city districts. If there were different classes present within the same cluster, the proportion between them was described frequently. Furthermore, the absence of classes was also part of cluster characterisation. Consequentially, defining constraints to preserve proportions between classes while maintaining at least one point per class is the second requirement.

#### Use Cartographic Techniques to Guide Interpretation

Beside density, the colour of points was the second most important factor influencing the task-solving strategies of the participants. As the use of specific colours to draw the map readers’ attention is a common technique in cartography, this can be adopted by point generalization algorithms. Especially with datasets where pattern preservation is difficult to manage, using style elements like colour and opacity could extend the variety of generalization techniques. Of course, any obvious thematic correlations for the specific applications have to be considered (such as keeping green colour for vegetation, if necessary). Furthermore, the Gestalt Laws regarding similarity and proximity should also be taken into account when using colors.

Another cartographic solution to help pattern recognition is an adjustment of the background map. As discussed in Sect. 5.3, the background map can have both an explicit and implicit influence on the user’s perception of patterns. Therefore, defining constraints for cartographic generalization which optimize the guiding effect of the background map (e.g. preservation of other map objects in close proximity to point clusters) could support the participant’s task-solving process.

## Conclusion

VGI often shows an enormous data volume in combination with semantic and temporal heterogeneity, often leading to undesired clutter effects. While existing cartographic generalization operations improve the readability of the map, specific spatial patterns in the data such as extreme values, clusters or hot spots, are rarely considered and can disappear through the generalization. To prevent users from being misled to false interpretations, map generalization constraints to maintain spatial pattern characteristics of point data are needed. To provide such a set of constraints, we first have to empirically analyse the user behaviour while interpreting point data sets.

We developed a new method, which adapted Think Aloud interviews and uses visualization techniques from eye-tracking analysis. Based on the results of our study, we can state that the point density of a dataset has the biggest impact on the user behaviour and the respective task-solving strategy, independently of the actual task type which is executed. We further examined the influences of point data cardinality and background map and identified only small impacts on the user behaviour when using background maps of different complexity.

Preparing the next step of our proposed research process, we further developed recommendations for constraint definition regarding the preservation of pattern proportions and the usage of cartographic techniques. Future work will further develop, implement and test respective constraints into generalization workflows. At this point, it could also be useful to conduct a follow-up study to the one presented here, focusing on a specific synoptic task with different complexity conditions and data sets. In addition, we will extend the focus of the present work: First, we want to apply our method to multiscale and multi-temporal displays. Second, we consider to broaden our work to more complex geometries, e.g. trajectories or buildings.

## Footnotes

1 https://www.psytoolkit.org/experiment-library/navon.html.

2 Our source code and an encoding example is online: https://gitlab.com/g2lab/think-aloud-visualizations.
